# Supplementary material for: Organ Specific Copy Number Variations in Visceral Metastases of Human Melanoma
Source: Cancers (Basel). 2021 Nov 28;13(23):5984. doi: 10.3390/cancers13235984 (PMC8657127; doi:10.3390/cancers13235984)
Supplement: Supplementary file 1 [file Figure S1.pdf › Figure S1.pdf]

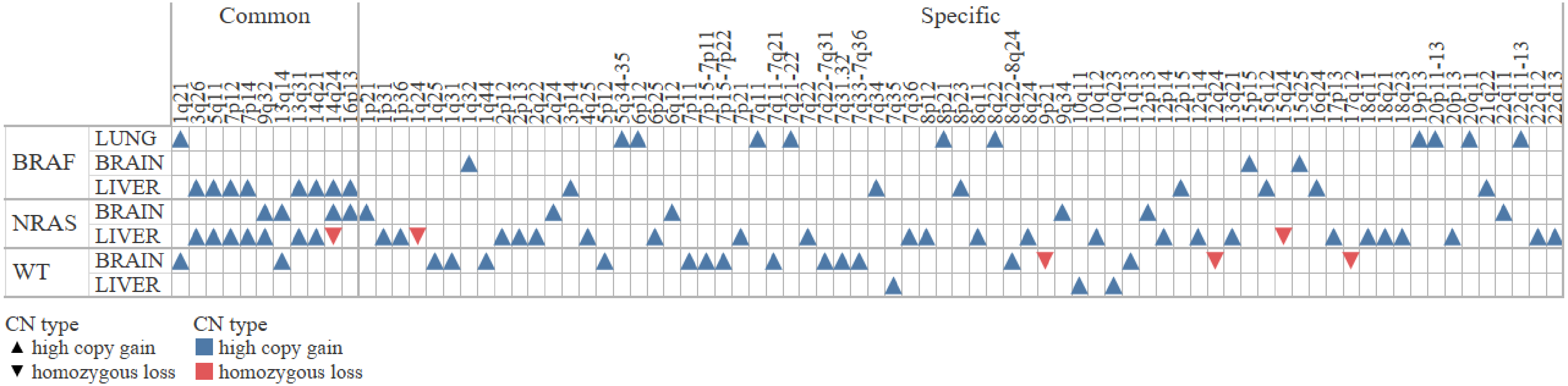

Figure S1: Shared and specific chromosomal regions distorted by high-copy number gains (CN≥4) and homozygous losses. Metastases were grouped based on their localization and divided into subgroups based on their detected driver mutations. Shared hCNG altered regions are 1q21 between BRAF mutant lungs and wild-type brains. NRAS mutant brain and liver mets has a common altered cytoband, 9p32. 14q24 was a homozygous lost chromosomal region in NRAS mutant livers, while NRAS mutant brains were modified in this cytoband by hCNG. NRAS mutant and wild type brains shared 13q14 hCNG. 3q26, 5q11, 7p12, 7p14, 13q31, 14q21 hCNGs were common features across BRAF and NRAS mutant liver metastases. The rest of the represented alterations were specific to different visceral sites depending on the driver mutation.
