# Supplementary material for: Organ Specific Copy Number Variations in Visceral Metastases of Human Melanoma
Source: Cancers (Basel). 2021 Nov 28;13(23):5984. doi: 10.3390/cancers13235984 (PMC8657127; doi:10.3390/cancers13235984)
Supplement: Supplementary file 1 [file cancers-13-05984-s001.zip › Table S2.pdf]

Table S2. Proportion of CNV types per genome in primary and distant metastatic melanomas.

tumor type	N	avg. % of genome altered ±*SE*		avg. % of CNG[1] ±*SE*		avg. % of hCNG[2] ±*SE*		avg. % of lCNG[3] ±*SE*	
		multiple group comparison	primary vs. all metastasis	multiple group comparison	primary vs. all metastasis	multiple group comparison	primary vs. all metastasis	multiple group comparison	primary vs. all metastasis
primary	10	13.37 ±*2.73*	13.37 ±*2.73*	10.08 ±*1.89*	10.08 ±*1.89*	0.09 ±*0.07*	0.09 ±*0.07*	9.99 ±*1.90*	9.99 ±*1.90*
brain	10	30.79 ±5.*14*		17.87 ±2.*59*		0.41 ±0.*21*		17.45 ±2.*62*	
liver	9	16.17 ±*4.46*	23.94 ±*2.92*	10.16 ±*2.77*	14.33 ±*1.72*	1.43 ±*0.79*	1.67 ±*0.54*	8.73 ±*2.32*	12.66 ±*1.63*
lung	9	24.10 ±*4.75*		14.58 ±*3.32*		3.30 ±*1.35*		11.28 ±*2.95*	
p-value[8]		0.056	**0.045**	0.179	0.214	**0.040**	**0.017**	0.113	0.507

tumor type	N	avg. % of CNL[4] ±*SE*		avg. % of hoCNL[5] ±*SE*		avg. % of heCNL[6] ±*SE*		avg. % of LOH[7] ±*SE*	
		multiple group comparison	primary vs. all metastasis	multiple group comparison	primary vs. all metastasis	multiple group comparison	primary vs. all metastasis	multiple group comparison	primary vs. all metastasis
primary	10	3.29 ±*1.04*	3.29 ±*1.04*	0.00 ±*0.00*	0.00 ±*0.00*	3.29 ±*1.04*	3.29 ±*1.04*	3.00 ±*0.30*	3.00 ±*0.30*
brain	10	12.93 ±2.*94*		0.07 ±0.*05*		12.85 ±2.*93*		8.10 ±1.*61*	
liver	9	6.02 ±*2.19*	9.61 ±*1.48*	0.02 ±*0.02*	0.04 ±*0.02*	6.00 ±*2.18*	9.58 ±*1.47*	12.31 ±*4.13*	10.60 ±*1.53*
lung	9	9.52 ±*2.03*		0.01 ±*0.01*		9.51 ±*2.02*		11.68 ±*1.75*	
p-value[8]		**0.021**	**0.019**	0.085	**0.012**	**0.021**	**0.019**	**0.003**	**0.0004**

[1]*copy number gain (both high- and low gains included)*

[2]*high copy number gain (CN ≥4)*

[3]*low copy number gain (2< CN <4)*

[4]*copy number loss (both homo- and heterozygous losses included)*

[5]*homozygous copy number loss (CN=0)*

[6] *heterozygous copy number loss (0< CN <2)*

[7] *loss of heterozygosity (copy neutral)*

[8] *Kruskal-Wallis (for multiple group analysis) or Mann-Whitney-Wilcoxon test (for primary vs. all metastsis analysis)*
