# Supplementary material for: Organ Specific Copy Number Variations in Visceral Metastases of Human Melanoma
Source: Cancers (Basel). 2021 Nov 28;13(23):5984. doi: 10.3390/cancers13235984 (PMC8657127; doi:10.3390/cancers13235984)
Supplement: Supplementary file 1 [file cancers-13-05984-s001.zip › Table S5.pdf]

Table S5. Proportion of melanoma samples (primary and melanoma mestastases) with HGF/MET/HGF+MET copy number gain in our melanoma cohort.

	N	HGF (%)	MET (%)	both (%)	correlation** of HGF&MET probe medians (p-value)
primary	10	3 (30.0)	3 (30.0)	3 (30.0)	0.115 (0.751)
brain	10	7 (70.0)	6 (60.0)	5 (50.0)	0.272 (0.446)
lung	9	7 (77.8)	3 (33.3)	3 (33.3)	0.699 (0.036)
liver	9	2 (22.2)	3 (33.3)	2 (22.2)	0.400 (0.286)
p*		*0.043*	*0.522*	*0.670*	

*Fisher's exact test, two-tailed (p≤0.05)

**Spearman's Rho correlation, two-tailed (p≤0.05)
