# Supplementary material for: Organ Specific Copy Number Variations in Visceral Metastases of Human Melanoma
Source: Cancers (Basel). 2021 Nov 28;13(23):5984. doi: 10.3390/cancers13235984 (PMC8657127; doi:10.3390/cancers13235984)
Supplement: Supplementary file 1 [file cancers-13-05984-s001.zip › Table S6.pdf]

Table S6: Representation of immunogenic mimicry related genes on transcriptomic and proteomic level.

Gene name	TCGA/mRNA	Protein	Protein/Au
CD1a/b/c/d	+	+	-
CD36	+	+	+
CD37	+	-	-
CD40	+	-	+
CD48	+	+	+
CD70	+	+	+
CD83	+	-	-
CD84	+	+	+
CD93	+	+	+
CD160	+	-	-
CD172	-	-	-
CD209	+	+	+
CD244	+	-	-
CD247	+	+	+
CD320	+	-	+
IDO1/2	+	+	+
BCR	+	+	+
IL17R	+	+	-

Column legend: TCGA/mRNA – mRNA expression data, Protein - prospective study cohort, Protein/Au – retrospective study cohort. If the given gene was found in one of the datasets then it is represented by „+” sign, while abscence is indicated by „-„ sign.
